# Reference genome and reproduction-focused transcriptome for the threatened alpine tree frog ( *Litoria verreauxii alpina*)

**DOI:** 10.12688/f1000research.163701.2

**Published:** 2025-09-02

**Authors:** Alexander S. Wendt, Laura Brannelly

**Affiliations:** 1 Department of Veterinary Sciences, The University of Melbourne Faculty of Science, Werribee, Victoria, 3030, Australia

**Keywords:** Anuran, Hylidae, genome assembly, conservation, Australia, threatened species

## Abstract

The alpine tree frog ( *Litoria verreauxii alpina*) is a threatened species found only above 1,200 meters within the Australian Alps. This species’ distribution has been severely limited due to the pathogentic amphibian chytrid fungus, and current populations persist by recruitment. Here, we provide the first publicly available genome for the genus. We used PacBio HiFi reads as well as Hi-C scaffolding data to construct a high-quality genome. We also generated a reproduction focused transcriptome from brain, liver, and gonad tissues. The genome was 2.77 Gb in length and consisted of 962 contigs with a contig N50 of 37.2 Mb and an L50 of 19. This study provides the first publicly available reference genome for the *Litoria* genus to assist in conservation and reproduction focused works in amphibian management.

## Introduction

**Figure 1.**
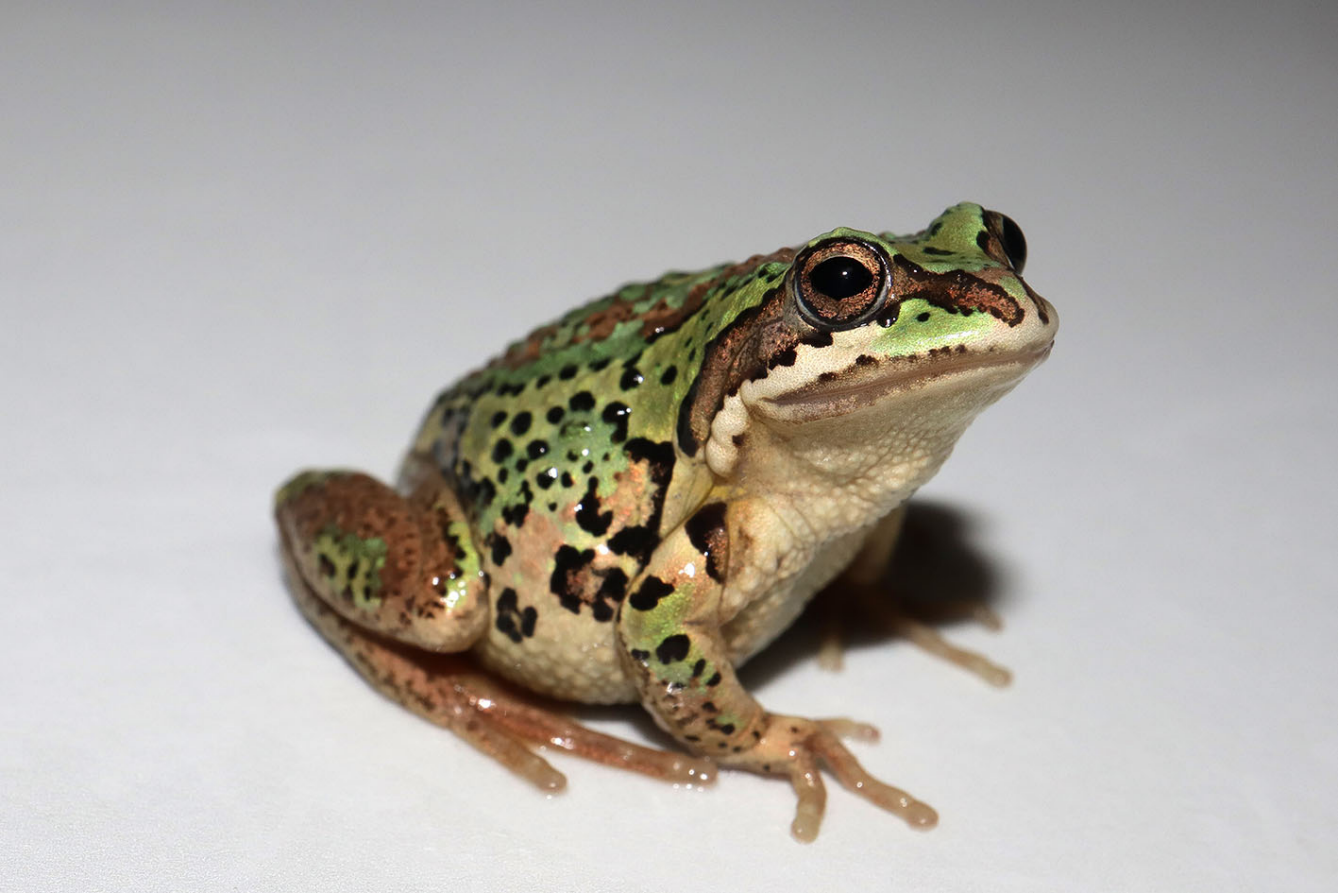
Photograph of a captive-bred alpine tree frog ( *Litoria verreauxii alpina*). Photo by Tiffany Kosch and Corey Doughty.

The alpine tree frog ( *Litoria verreauxii alpina*; Figure 1) is endemic to the Australian Alps of New South Wales and Victoria, occurring at elevations above 1,200 meters ( Brannelly *et al.*, 2015). Since the introduction of the pathogenic amphibian chytrid fungus *Batrachochytrium dendrobatidis* ( *Bd*) to Australia, the species’ distribution has declined by over 80% since the 1980s, leaving only a few remaining populations ( Gillespie, Osborne, & McElhinney, 1995; Hunter, Osborne, & Smith, 1998; Osborne, Hunter, & Hollis, 1999; Hunter *et al.*, 2009). Adult *L. v. alpina* are highly susceptible to *Bd* infection, with prevalence rates approaching 100% during the breeding season ( Brannelly *et al.*, 2015; Scheele *et al.*, 2015). The species exhibits minimal protective immunity against the disease, leading to near-complete population turnover each breeding cycle ( Bataille *et al.*, 2015; Grogan *et al.*, 2018; Brannelly *et al.*, 2015; Scheele *et al.*, 2015).

Despite these challenges, the remaining *L. v. alpina* populations persist, largely due to a compensatory reproductive strategy. Infected individuals exhibit increased reproductive effort, as evidenced by larger gonadal structures and higher gamete production compared to uninfected counterparts ( Scheele *et al.*, 2015; Brannelly *et al.*, 2016, 2021, 2025). This strategy may help offset high mortality rates, ensuring continued recruitment despite the overwhelming impact of *Bd.* However, the long-term effectiveness of this response remains uncertain, particularly as other environmental pressures further threaten population stability. Understanding the genetic mechanisms underlying this reproductive adaptation is crucial for assessing the species’ resilience and informing conservation strategies.

The *Litoria* genus, to which *L. v. alpina* belongs, is highly diverse, comprising over 150 recognized species across Australia (‘ AmphibiaWeb’, 2025). Despite its ecological and evolutionary significance, no publicly available reference genomes for any species within the genus were identified in the Australian Reference Genome Atlas (ARGA) as of February 25, 2025 ( Hall *et al.*, 2023). To address this gap, we generated a high-quality reference genome for *L. v. alpina*, along with a reproduction-focused transcriptome derived from tissues critical to reproductive function which included the brain, liver, and gonads. These genomic resources provide a foundation for investigating genetic variation within the species, shedding light on how *L. v. alpina* maintains population persistence in the face of extreme disease pressure. Additionally, this work fills a critical gap in genomic knowledge within *Litoria*, offering new opportunities to study evolutionary relationships, reproductive adaptations, and broader ecological dynamics across the genus.

## Methods

### Sample collection and DNA/RNA extraction

Samples were collected from two adult males and one adult female *L. v. alpina* that were lab-raised from eggs at the University of Melbourne, Werribee campus, Victoria, Australia ( Brannelly, Sharma, & Wallace, 2023). The individuals sampled were part of a larger experiment that involved humane euthanasia as the endpoint. Individuals were medically euthanized via immersion for ≥10 min in 3 mL of 100 mg/L tricaine methanesulfonate (MS-222) buffered with sodium bicarbonate. Individuals were removed from the MS-222 solution after becoming unresponsive and immediately decapitated (University of Melbourne’s Animal Ethics application: 26083). Tongue, muscle from the right thigh, and liver tissue were removed from one male (Lva_1) while brain, liver, and gonads (testes or ovaries) were extracted from the other male and female individual (Lva_2 and Lva_3 respectively). All samples were flash frozen using liquid nitrogen and stored at -80°C until extraction.

High molecular weight (HMW) DNA was extracted from the tongue and muscle tissue of Lva_1 using the Monarch® HMW DNA Extraction Kit for Cells & Blood (New England Biolabs: T3050S) following the manufacturer protocols. Concentrations and quality were then assessed via a Femto Pulse genomic DNA 165 kb kit (Agilent: FP-1002-0275), Qubit™ dsDNA BR assay kit (Thermo Fisher Scientific; Table 1), and NanoDrop (Thermo Fisher Scientific; Table 1), with the highest yielding sample used for library preparation.

**Table 1.** HMW DNA concentrations and purity measurements for muscle and tongue tissue from individual Lva_1.

Sample	Qubit (ng/μL)	Nanodrop (ng/μL)	260/280	260/230
Lva_1_Muscle	11.6	19.4	1.62	0.86
Lva_1_Tongue	376	178.1	1.79	1.88

Total RNA was extracted from the brain, liver, and gonad tissues collected from Lva_2 and Lva_3 individuals using the RNAeasy Plus Mini Kit (Qiagen: 74134) with RNAse-free DNAse I set (Qiagen: EN0521) digestion. RNA quantity was determined using a Qubit 3 fluorometer with an Invitrogen™ Qubit RNA High Sensitivity Kit (Thermo Fisher Scientific) and RNA integrity (RIN) score determined using a 5200 Fragment Analyzer (Agilent; Table 2).

**Table 2.** RNA concentrations and quality scores for brain, liver, and gonad samples from male (Lva_2) and female (Lva_3) *Litoria verreauxii alpina.*

Sample	Concentration (ng/μL)	Quality Score (RIN)	Total (ng)	DV200 (%)
Lva_2_Brain	25.8	9.6	801	90
Lva_2_Liver	16.5	9.9	512	88
Lva_2_Testes	56.3	10.0	1746	93
Lva_3_Brain	14.0	9.8	433	86
Lva_3_Liver	4.2	-	129	66
Lva_3_Ovary	23.8	9.9	738	91

### Library construction and sequencing

The HMW DNA from Lva_1 tongue tissue was sent for Pacific Biosciences High Fidelity (PacBio HiFi) library preparation with a SMRTbell® prep kit 3.0 (Pacific Biosciences: 102-141-700) and Revio™ polymerase kit (Pacific Biosciences: 102-739-100) and sequencing on one single molecule real-time (SMRT) cell on a PacBio Revio at Australian Genome Research Facility (AGRF), Brisbane, Australia.

Two LinkPrep libraries were prepared from liver tissue from Lva_1 using the Dovetail® LinkPrep™ Kit (Cantata Bio) at the Advanced Genomics Services of the Australian Genome Research Facility. Briefly, the chromatin was fixed with disuccinmidyl glutarate (DSG) and formaldehyde in the nucleus. The cross-linked chromatin was then fragmented and tagged with Tn5 transposase in situ. Next, the cells were lysed to extract the chromatin fragments, which were subsequently bound to chromatin capture beads. Proximity ligation was then performed, whereby chromatin fragments that were in proximity to one another were ligated together. After proximity ligation, the crosslinks were reversed, the associated proteins were degraded, and the DNA was purified and converted into a sequencing library. Each library was sequenced on an Illumina Novaseq X plus platform to generate 2 million 2 × 150 bp read pairs to assess the quality of mapping, valid *cis* - *trans* reads and complexity of the library. For chromosome level assembly, each library was sequenced approximately 100 million 2 × 150 bp read pairs per Gb of the genome size at the Australian Genome Research Facility, Melbourne, Australia.

Total RNA from the brain, liver, and gonads of individuals Lva_2 and Lva_3 was prepared using Illumina Total RNA with RiboZero Plus library preparation and sequenced as 150 bp paired-end reads on an Illumina NovaSeq 6000 at the Australian Genome Research Facility, Melbourne, Australia.

### Genome assembly

Genome assembly was conducted on the Galaxy Australia platform using workflows developed by Bioplatforms Australia Threatened Species Initiative, Galaxy Australia, and the Australian BioCommons ( https://australianbiocommons.github.io/how-to-guides/). After the upload of the raw HiFi reads in.ccs.bam format provided by AGRF, we used the BAM to FASTQ + QC v1.0 workflow ( Price, 2022 *a*). This utilizes SamtoFastq v2.18.2.2 ( Broad Institute, 2009), Samtools flagstat v2.0.3 ( Danecek *et al.*, 2021), and FastQC v0.72 ( Andrews, 2010). Following file conversion, we ran the PacBio HiFi genome assembly using hifiasm v2.1 workflow ( Price & Farquharson, 2022). This process produced a draft genome assembly in FASTA format, accompanied by assembly metrics and a detailed report. HiFi reads underwent adapter sequence removal using HiFiAdapterFilt v2.0.0 ( Sim *et al.*, 2022), followed by de novo assembly using hifiasm v0.16.1 ( Cheng *et al.*, 2021). To evaluate assembly structure and completeness, the assembly graph was visualized using Bandage Image v0.8.1 ( Wick *et al.*, 2015). Bandage Info v0.8.1 was used to extract key assembly statistics, such as contig N50 and total assembly length, providing insights into the overall quality of the assembly.

To enhance the assembly’s accuracy, the purge duplicates from hifiasm assembly v1.0 workflow ( Price, 2022 *b*) was applied to remove haplotype repeats. This step uses minimap2 v2.28 ( Li, 2018) and purge_dups v1.2.6 ( Guan *et al.*, 2020) to align and purge duplicates based on read depth. We then scaffolded the Hi-C reads with the genome using the TSI scaffolding with HiC (based on VGP-HiC-scaffolding) v1.0 workflow ( Syme & Silver, 2024). The scaffolding workflow utilizes several tools including BWA-MEM2 v2.2.1 ( Li & Durbin, 2010; Li, 2013), YAHS v1.21.2 ( Zhou, McCarthy, & Durbin, 2023), gfastats v1.3.10 ( Formenti *et al.*, 2022), bedtools BAM to BED v2.31.1 ( Quinlan & Hall, 2010), and PretextMap v0.1.9 ( Harry, n.d.). The finished genome was assessed using the genome assessment post assembly workflow ( Farquharson *et al.*, 2024), which produces Fasta statistics v2.0, Quast v5.0.2 ( Mikheenko *et al.*, 2018), BUSCO v5.4.6 ( Simão *et al.*, 2015), and Merqury v1.3 ( Rhie *et al.*, 2020) outputs.

### Transcriptome assembly

Transcriptome assembly was also conducted on the Galaxy Australia platform using workflows developed by Bioplatforms Australia Threatened Species Initiative. To minimize interference from repetitive genomic elements, the reference genome was first subjected to repeat masking using the Repeat Masking v3.0 workflow ( Silver & Syme, 2024 *a*). The workflow processed the reference genome FASTA file, generating both hard-masked and soft-masked genome files along with a statistics report detailing the extent of masking. Quality control and adapter trimming of raw RNA sequencing reads were performed using the QC and Trimming of RNAseq Reads v1.0 workflow ( Silver & Syme, 2024 *b*) for each tissue separately. This step involved filtering low-quality bases and removing sequencing adapters. Trimmomatic Galaxy v0.36.6 was used to trim-reads specifying NEXTERA (pair-ended) adapters, SLIDING-WINDOW:4:5, LEADING:5, TRAILING:5 and MINLEN:25 ( Bolger, Lohse, & Usadel, 2014). The soft repeat-masked genome was indexed and reads were aligned using HiSAT2 v2.2.1 ( Kim *et al.*, 2019). Quality was assessed using FASTQC v0.74 ( Andrews, 2010), and the processed reads were retained as paired FASTQ files for subsequent analysis.

Processed RNA-Seq reads were aligned by tissue and individual of origin to the soft-masked reference genome using the Align Reads to Find Transcripts v1.0 workflow ( Silver & Syme, 2024 *c*). This alignment generated BAM and GTF files, providing transcript structures and alignment metrics to aid in genome annotation. Transcriptome assembly was conducted using the Combine Transcripts v1.0 workflow ( Silver & Syme, 2024 *d*), which integrated tissue-specific transcript data into a comprehensive global transcriptome. Coding sequences were predicted based on sequence homology with *Xenopus laevis* coding DNA (cDNA) downloaded from NCBI. The workflow output included a GTF file representing the global transcriptome and FASTA sequences of coding transcripts.

To identify the longest isoforms, the Extract Longest Transcripts v1.0 workflow ( Silver & Syme, 2024 *e*) was applied. TransDecoder was used to predict coding sequences, filtering transcripts to retain only the longest isoform per gene. The resulting outputs included peptide FASTA files, coding sequence FASTA files, and GFF3 annotation files for further analyses. The final step involved converting the transcriptome annotation outputs into formats compatible with genome annotation tools. The Convert Outputs v1.0 workflow ( Silver & Syme, 2024 *f*) was used to process TransDecoder peptide FASTA files and global nucleotide FASTA files into .cdna, .dat, and .pro formats required for downstream annotation applications.

### Genome annotation

Genome annotation was performed using the FgenesH++ tool on the Galaxy Australia platform using the assembled reference genome, the hard-masked genome and the.cdna, .pro, and.dat files generated by the Convert Outputs v1.0 ( Silver & Syme, 2024 *f*) workflows as input files. The Fgenesh annotation v3.0 workflow ( Silver, 2024) was executed, which involves genome splitting, annotation, merging of annotation files, and extraction of mRNA, CDS, and protein sequences. The settings used the *Xenopus* (generic frog) gene-finding matrix and a non-mammalian database. The outputs included GFF3 files of annotated genes and FASTA files for mRNA, CDS, and protein sequences. BUSCO v5.4.6 in ‘protein’ modes was used to assess the annotation with the tetrapoda_odb10 lineage.

## Results

### Genome size and assembly

**Figure 2.**
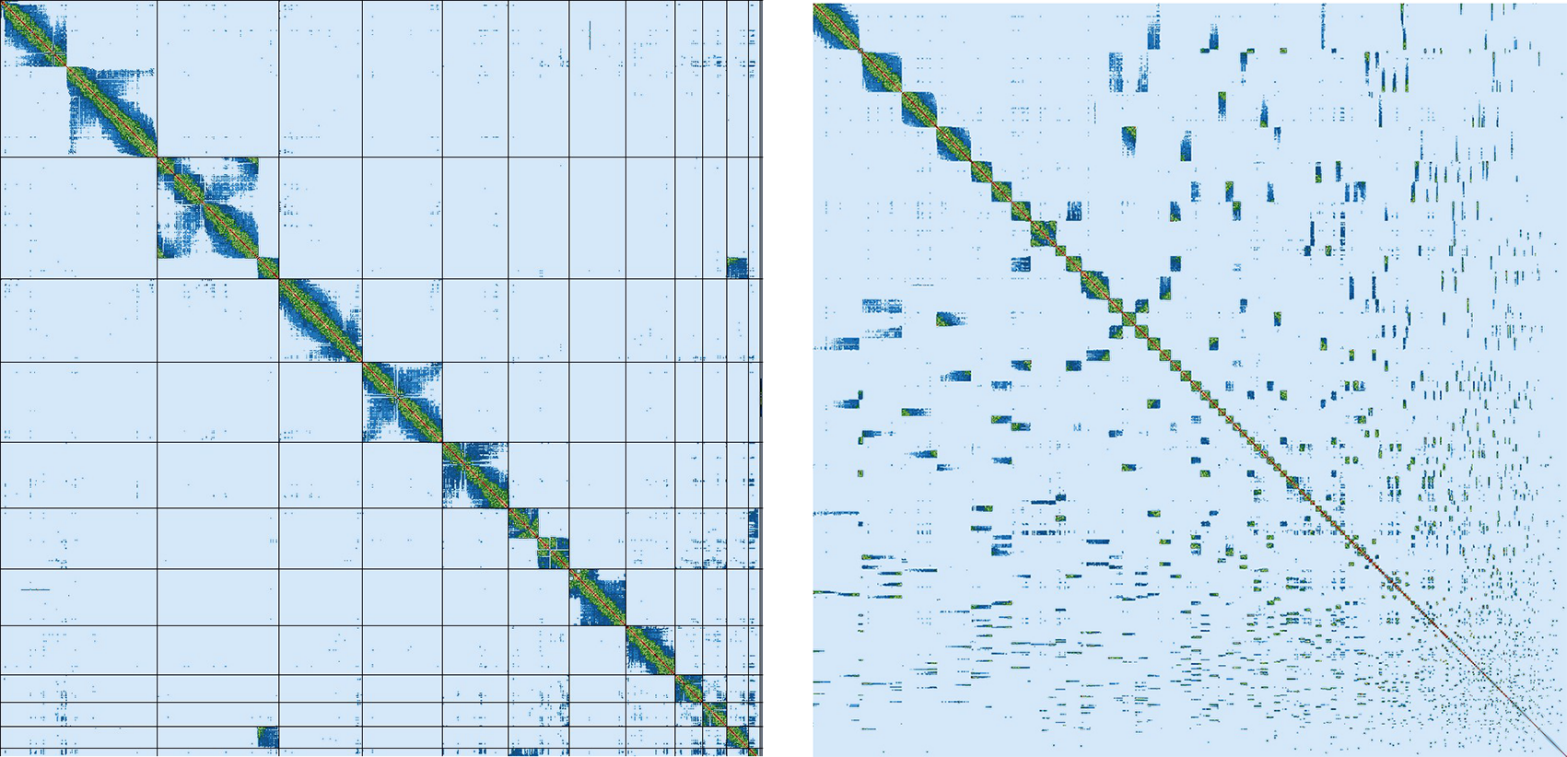
Hi-C contact map of the *L.v. alpina* reference genome. Left: represents contacts for the first 12 scaffolds of the genome. Right: represents contacts for all 774 scaffolds of the genome. Scaffolds are shown in order of size from top-left going right at a diagonal.

Assembly of the male *Litoria verreauxii alpina* resulted in a genome of 2.77 Gb, which was comprised of 962 contigs with a contig N50 of 37.17 Mb. The genome was sequenced using PacBio HiFi reads which generated 87.92 Gb from 7,764,356 reads, resulting in a coverage of 31.74×. Primary assembly contigs were scaffolded using proximity-based enrichment Hi-C data ( Figure 2), which produced 248.99 Gb from 829,962,675 reads. Genome scaffolding with this data resulted in 774 scaffolds with 188 gaps and a scaffold N50 of 267.09 Mb ( Table 3). The majority (91.3%) of the assembly mapped to the first 13 scaffolds, reflecting the 13 chromosome karyotype described for the species ( Schmid *et al.*, 2018) and within other *Litoria* species ( Ferro *et al.*, 2018; Mollard, Mahony, & West, 2024; Kosch *et al.*, 2025).

**Table 3.** Genome assembly statistics for the *Litoria verreauxii alpina* genome.

Genome assembly	
Assembly length	2,772,442,494
Number of contigs	962
Contig N50	37,168,586
Contig L50	19
Contig N90	2,484,530
Contig L90	130
Longest contig	169,435,372
Number of scaffolds	774
Scaffold N50	267,093,197
Scaffold L50	4
Scaffold N90	36,543,458
Scaffold L90	12
Longest scaffold	522,534,866
GC content %	43.04
Read coverage	31.74

**Figure 3.**
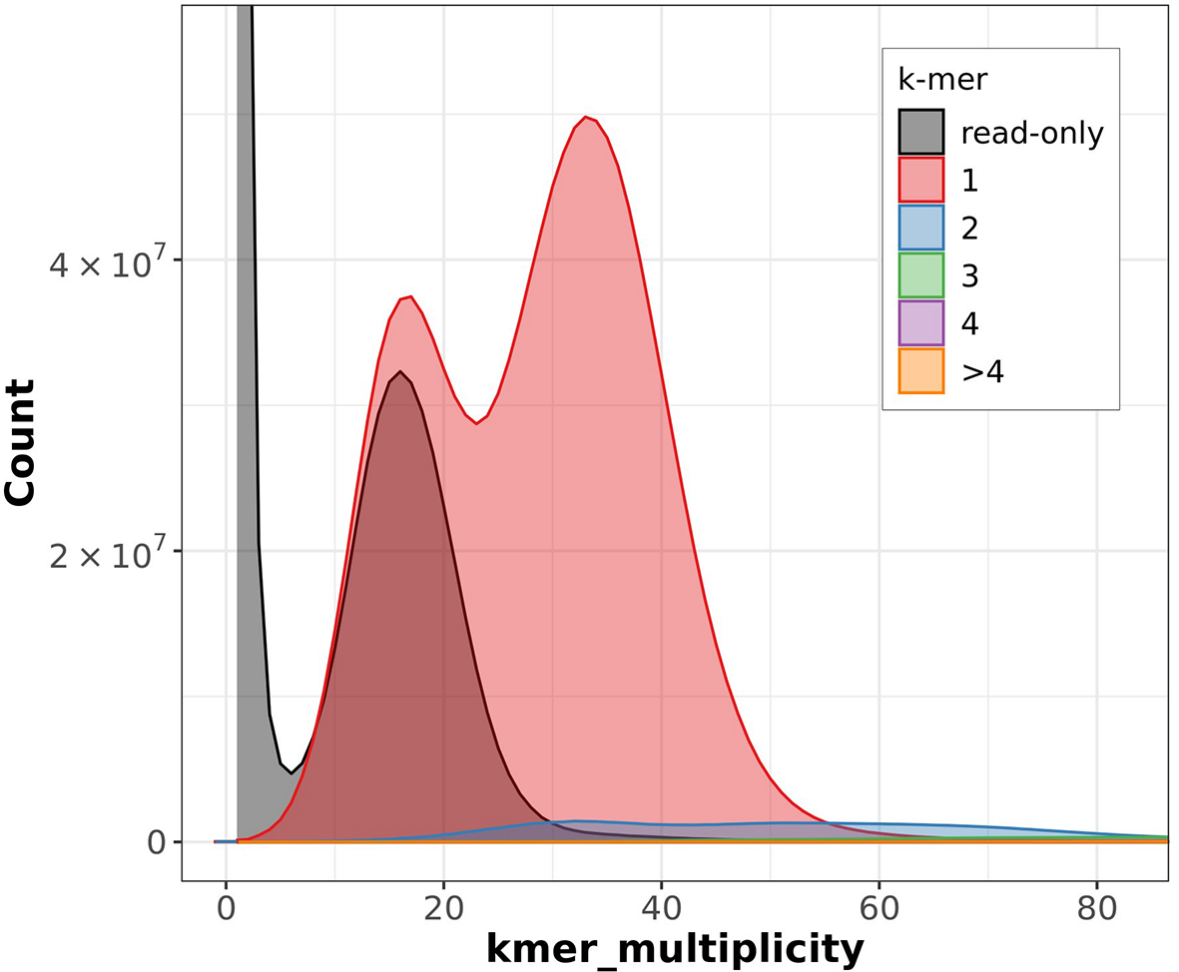
Merqury output showing the copy number of k-mers. ‘k-mer multiplicity’ records the number of times a certain k-mer appears in the reads, and ‘Count’ records the number of k-mers that have appeared that number of times. Grey represents the k-mers found only in the reads, while the colors correspond to the number of k-mers that have appeared at that given number of times.

The Merqury estimated Quality Value (QV) of the final assembly was 63.7 with an error rate of 4.2e ^−7^. Completeness with Merqury was lower than expected at 78.6%, which is most likely due to the fact that the purge duplicates workflow removed a high number of repetitive sequences and haplotigs ( Figure 3). Before purging, the genome was 2777517237 bp and had a 100% completeness score. After purging, the genome was reduced to 2772442494 bp with most of the purged sequences labeled as high coverage, haplotig, or repeat sequences. BUSCO v5.4.6 indicated a completeness of 90.1% (single = 86.7%, duplicate = 3.4%), using the tetrapoda_odb10 reference set (n = 5310) ( Table 4).

**Table 4.** Genome assembly metrics of the completed *Litoria verreauxii alpina* genome.

Genome assembly metrics	
Quality value (QV) *	63.7 (4.2e ^−7^)
Completeness	78.6%
BUSCO **	C:90.1% [S:86.7%, D:3.4%], F:2.9%, M:7.0%, n:5310

* Consensus quality value (error rate).

** BUSCO scores based on the tetrapoda_odb10 BUSCO reference set using version 5.4.6. C = complete, S = single copy, D = duplicated, F = fragmented, M = missing, n = number of orthologues in comparison.

### Transcriptome assembly and genome annotation

After quality trimming, 99.48% of reads were retained. Individual tissues had a high number of duplicate reads ranging from 57.2% – 85.0%. The individual tissue transcriptomes had varying mapping rates to the soft repeat-masked genome (78.77% female brain; 77.89% male brain; 80.59% female liver; 83.50% male liver; 80.92% ovary; 81.59% testes). A total of 98760 transcripts were used as evidence for the genome annotation. Repetitive elements comprised 61.43% of the total genomic sequence, with 41.88% of these consisting of unclassified repeats. A total of 40092 genes were predicted from the annotation ( Table 5). There was an average of 6.2 exons (SE=34.6) per putative gene with an average exon length of 229 bp (SE=556) and an average intron length of 3353 bp (SE=12458). The reproduction focused annotation had 65.4% BUSCOs [Single copy: 62.8%; Duplicated: 2.6%]; 14.2% fragmented BUSCOs and 20.4% missing BUSCOs.

**Table 5.** Reproduction focused genome transcriptome statistics, repeat content, and alignment of the *Litoria verreauxii alpina* genome.

Genome annotation statistics				
	% of genome	Average size (bp)	Median size (bp)	n
Exon	2	229	126	249568
Gene	28	19299	7699	40092
Intron	25	3353	1033	209476

Genome repeat content		
Repeat element	Number of elements	% of genome
DNA transposons	867221	9.69
LINEs	444639	5.79
SINEs	31630	0.23
LTRs	386024	3.84
Simple	81751	0.14
Unclassified	6153713	41.88
Total interspersed repeats		61.43
Small RNA	50566	0.46
Satellites	5903	0.13
Simple repeats	81751	0.14

## Ethical considerations

Frogs were humanely euthanized following the completion of previous experimental procedures under the University of Melbourne (Victoria, Australia) Animal Ethics permit #26083.

## Data Availability

The raw PacBio HiFi, Omni-C, and RNA read data is publicly available from NCBI’s Short Read Archive (SRA) accession numbers: SRR32377441, SRR32377442, SRR32314942-SRR32314944, SRR32314946, SRR32581849, SRR32581850 ( Wendt & Brannelly, 2025a). And the assembled genome is available on NCBI’s Assembly database, BioProject: PRJNA1219307 ( Wendt & Brannelly, 2025b). The Arrive Author Checklist can be found on the University of Melbourne Figshare: Author Checklist – ARRIVE.pdf, HYPERLINK https://doi.org/10.26188/28899941.v2 ( Wendt & Brannelly, 2025c). Data are available under the terms of the Creative Commons Zero “No rights reserved” data waiver (CC0 Public domain dedication).
