# Supplementary material for: Reference genome and reproduction-focused transcriptome for the threatened alpine tree frog ( Litoria verreauxii alpina)
Source: F1000Res. 2025 Sep 2;14:514. Originally published 2025 May 23. [Version 2] doi: 10.12688/f1000research.163701.2 (PMC12171712; doi:10.12688/f1000research.163701.2)
Supplement: Peer review history

# Reviewer report: Reviewer response for version 1

**DOI:** 10.5256/f1000research.180093.r387736

**Published:** 2025-06-16

**Authors:** Martin Knytl

**Affiliations:** 1 Charles University, Viničná, Czech Republic

The proposed manuscript “Reference genome and reproduction-focused transcriptome for the threatened alpine tree frog (Litoria verreauxii alpina)” presents a valuable de-novo genome assembly at the scaffold level for this threatened species. One of the key fundings is the mapping of over 90% of reads to the 13 largest scaffolds, leading the authors to propose these to represent 13 chromosomes in the karyotype of L. v. alpina. The manuscript contributes to genomics, offering a usable and reproducible resource - the genome assembly is already deposited in the NCBI database. I found no fundamental issues within the proposed manuscript. It is technically sound and written in high-quality English. Furthermore, all methods and results pertaining to the genome and transcriptome analyses are presented with clarity, and the text is well-structured, creating a coherent narrative.

 I have only minor comments/questions to further enhance the manuscript's clarity:

 1) Species Description: The introduction could benefit from a more detailed description of the species L. v. alpina. Specifically, including its taxonomic classification (e.g., the family level, batrachia subgroup) and relevant phylogenetic context would be valuable for readers less familiar with this organism.

 2) Scaffold Count and Chromosome Number: The authors propose that the 13 largest scaffolds likely represent the 13 chromosomes. However, Table 3 indicates an L90 of 12 scaffolds. Clarification would be beneficial as to whether the L90 value of 12 scaffolds has any bearing on the chromosomal count (and if Table 3 might have a typo), or if it's an independent assembly metric.

Are the datasets clearly presented in a usable and accessible format, and the assembly and annotation available in an appropriate subject-specific repository?

Yes

Are sufficient details of the sequencing and extraction, software used, and materials provided to allow replication by others?

Yes

Are the rationale for sequencing the genome and the species significance clearly described?

Yes

Are the protocols appropriate and is the work technically sound?

Yes

Reviewer Expertise:

Chromosome evolution, Sex chromosomes, Polyploidy, Cytogenetics, Evolutionary genomics, Sanger sequencing, Genome editing, Cytogenomics

I confirm that I have read this submission and believe that I have an appropriate level of expertise to confirm that it is of an acceptable scientific standard.

# Reviewer report: Reviewer response for version 1

**DOI:** 10.5256/f1000research.180093.r387730

**Published:** 2025-06-06

**Authors:** Yanbo Sun

**Affiliations:** 1 Yunnan University, Kunming, China

Summary of the Article

 The study presents the first reference genome (2.77 Gb) and a reproduction-focused transcriptome for the threatened alpine tree frog (Litoria verreauxii alpina), a species endemic to the Australian Alps. The genome was assembled using PacBio HiFi reads (31.74× coverage) and scaffolded with Omni-C proximity ligation data. Transcriptomes were generated from brain, liver, and gonad tissues of male and female frogs. The genome assembly yielded 774 scaffolds with a scaffold N50 of 267.09 Mb, and annotation predicted 40,092 genes. The work aims to support conservation efforts by elucidating genetic mechanisms behind the species' compensatory reproductive strategy, which offsets high mortality from chytridiomycosis.

 While data accessibility and rationale are exemplary, methodological opacity and technical inconsistencies must be resolved to ensure replicability and scientific rigor. 

 Insufficient parameter details: Assembly tools (e.g., hifiasm, minimap2, purge_dups) lack runtime parameters, k-mer sizes, or filtering thresholds.

 BUSCO vs. Merqury discrepancy: The genome completeness dropped from 100% (pre-purge) to 78.6% (post-purge) in Merqury, while BUSCO reported 90.1%. Authors must reconcile this by analyzing purged sequences (e.g., whether critical genes were removed).

 Software versions are provided (e.g., hifiasm v0.16.1), but critical parameters (e.g., --purge-dups thresholds, Hi-C scaffolding stringency) are omitted.

 RNA-Seq trimming parameters are noted, but adapter sequences and quality-filtering cutoffs are not specified.

 Ethical permit details lack a link to institutional guidelines or approval documentation.

Are the datasets clearly presented in a usable and accessible format, and the assembly and annotation available in an appropriate subject-specific repository?

Yes

Are sufficient details of the sequencing and extraction, software used, and materials provided to allow replication by others?

Partly

Are the rationale for sequencing the genome and the species significance clearly described?

Yes

Are the protocols appropriate and is the work technically sound?

Partly

Reviewer Expertise:

Evolutionary genomics of amphibians and reptiles

I confirm that I have read this submission and believe that I have an appropriate level of expertise to confirm that it is of an acceptable scientific standard.

# Response

**Published:** 2025-06-09

**Authors:** Alexander Wendt

**Affiliations:** Veterinary, The University of Melbourne Faculty of Science, Melbourne, Victoria, Australia

We found that the default parameters within the Galaxy workflows for assembly (e.g., hifiasm, minimap2, purge_dups) were sufficient for our assembly, and adjusting these parameters made no noticeable difference to the final output (several test runs with other parameters were performed). Specifically:

- **hifiasm**: default -l 44, -m 97, **hifiasm** determines k-mer sizes automatically during assembly, based on the input read characteristics (such as read length and coverage). No explicit k-mer sizes were set in our workflow.
- **minimap2**: default -N 5, -F 800, -f 0.0002, -g 5000, -r 500, -n 3, -m 40, -p 0.8,
- **purge_dups**: default thresholds
- **YaHS Hi-C scaffolding**: default settings, including:
  - Mapping quality threshold (-q): 10
  - Contig and scaffold error correction: both enabled by default.

These default settings provided moderate stringency, balancing accuracy and scaffold continuity. We chose to retain these default parameters to ensure easy reproduction of the workflows and minimize potential biases from over-optimization. These parameters are also associated with the version of the workflow and can be referenced accordingly. We thank the reviewer for highlighting this point and are happy to provide these details here to improve transparency.

 While genome completeness measured by Merqury dropped from 100% to 78.6% post-purge, the BUSCO score remained unchanged. This discrepancy likely reflects the high repetitive content typical of amphibian genomes, as these repetitive elements and high copy-number regions are over represented during initial sequencing and are more susceptible to removal during purging. In contrast, BUSCO targets single-copy orthologs, which remain stable regardless of repeat content. Scaffold 1 was mainly affected during the purging of duplicates and the genome was reduced by 5,074,743 bp.

 For RNA-Seq trimming, this was specified in the manuscript: Trimmomatic Galaxy v0.36.6 was used to trim reads specifying NEXTERA (pair-ended) adapters, SLIDING-WINDOW:4:5, LEADING:5, TRAILING:5, and MINLEN:25

 Regarding the ethical permit details, we have submitted the official approval letter to the journal during the manuscript submission process. While this letter is not intended for public dissemination, it has already been vetted by the journal’s editorial office.

 Once again, we thank the reviewer for their thoughtful comments and for highlighting these areas for clarification. We believe these additional details will strengthen the manuscript and improve transparency for future readers
